# Targeting lactate dehydrogenase a improves radiotherapy efficacy in non-small cell lung cancer: from bedside to bench

**DOI:** 10.1186/s12967-021-02825-2

**Published:** 2021-04-26

**Authors:** Yang Yang, Yu Chong, Mengyuan Chen, Wumin Dai, Xia Zhou, Yongling Ji, Guoqin Qiu, Xianghui Du

**Affiliations:** 1 Department of Thoracic Radiotherapy, Cancer Hospital of the University of Chinese Academy of Sciences (Zhejiang Cancer Hospital), Hangzhou, 310022 China; 2 Institute of Cancer and Basic Medicine (IBMC), Chinese Academy of Sciences, Hangzhou, 310022 China; 3 State Key Laboratory of Radiation Medicine and Protection, School for Radiological and Interdisciplinary Sciences (RAD-X), and Collaborative Innovation Center of Radiation Medicine of Jiangsu Higher Education Institutions, Soochow University, Suzhou, 215123 China; 4 Department of Clinical Lab, Cancer Hospital of University of Chinese Academy of Sciences, Hangzhou, 310022 China; 5 Zhejiang Key Laboratory of Radiation Oncology, No 1, East Banshan Road, Gongshu District, Hangzhou, 310022 People’s Republic of China

**Keywords:** LDHA, NSCLC, Radiosensitivity, DNA repair, ROS, Apoptosis

## Abstract

**Background:**

Lactate dehydrogenase A **(**LDHA) is overexpressed and associated with poor prognosis in many kinds of cancer. In the current study, we evaluated the prognostic value of LDHA expression in non-small cell lung cancer (NSCLC), and tested whether LDHA inhibition might improve radiotherapy efficacy in NSCLC.

**Methods:**

LDHA expression was investigated in NSCLC patients, using online database and further verified by immunohistochemistry. The prognostic value of LDHA was evaluated using Kaplan–Meier plotter database. In vitro, two NSCLC cell lines were pretreated with oxamate, an inhibitor of LDHA, and colony formation method was performed to determine cellular radiosensitivity. Comet assay was used to detect DNA damage after irradiation. Flow cytometry was applied to test cell cycle progression and apoptosis, and monodansylcadaverine (MDC) staining was used to examine cell autophagy.

**Results:**

Both mRNA and protein levels of LDHA expression were up-regulated in NSCLC tissues. High LDHA expression was a poor prognostic factor and associated with radioresistance in NSCLC patients. LDHA inhibition by oxamate remarkably increased radiosensitivity in both A549 and H1975 cancer cells, and enhanced ionizing radiation (IR)-induced apoptosis and autophagy, accompanied by cell cycle distribution alternations. Furthermore, LDHA inhibition induced reactive oxygen species (ROS) accumulation and cellular ATP depletion, which might increase DNA injury and hinder DNA repair activity.

**Conclusions:**

Our study suggests that inhibition of LDHA may be a potential strategy to improve radiotherapy efficacy in NSCLC patients, which needs to be further tested by clinical trials.

## Background

Lung cancer is the most common malignancy, making up almost 25% of all cancer deaths around the world [1]. Radiotherapy plays a vital role in lung cancer treatment, almost three quarters of all lung cancer patients need to receive radiotherapy at some point in their lives, and concurrent chemo-radiotherapy is now the cornerstone of curative treatment for unresectable locally advanced lung cancer [2]. However, owing to the existence of endogenous radio-resistance, which often leads to local failure and tumor progression, the outcomes of stage III patients after CRT is still disappointed, with a 5-year survival rate of 15–20% approximately. Although emerging immunotherapy has further improved the survival recently [3]. There is still an urgent need to seek for novel strategies to enhance radiotherapy efficiency in NSCLC patients.

High metabolism has been recognized as a new hallmark of cancer, and emerged as a potential target for anti-cancer drugs [4]. As a key enzyme evolved in glycolysis, LDHA has been reported to be up-regulated and greatly associated with poor prognosis in many kinds of cancer [5–9]. Accumulating evidence indicates that LDHA inhibition not only exhibits promising anti-cancer effects through disrupting cellular energy metabolism, but also synergistically enhances the efficacy of other therapeutic regimens, including chemotherapy [10], radiotherapy [11, 12] and target drugs [13–15]. We previously reported that diverse biological effects were observed in NSCLC cells induced by LDHA inhibition, including autophagy or apoptosis, accompanied with different cell cycle distribution alternations [16]. However, the role of LDHA in regulating radiosensitivity of NSCLC has not been elucidated up to now.

In this study, we explored the mRNA expression of LDHA in NSCLC patients using online database, and further verified by immunohistochemistry (IHC). Then, the prognostic value of LDHA in NSCLC was evaluated using Kaplan–Meier plotter database. To explore the effect of LDHA inhibition on the radiosensitivity of NSCLC, two NSCLC cell lines (A549 and H1975 cells) were pretreated with oxamate, one widely used inhibitor of LDHA as a structural analogue of pyruvate, and followed with the exposure to different doses of irradiation. In addition, the possible underlying mechanism was also investigated.

## Methods

### Online database

Levels of LDHA mRNA expression in NSCLC tumor and normal tissue were compared by GEPIA browser (http://gepia.cancer-pku.cn/), a newly developed interactive web server for analyzing the RNA sequencing expression data of 9736 tumors and 8587 normal samples from the TCGA and the GTEx projects, which provides customizable functions such as tumor/normal differential expression analysis, profiling according to cancer types or pathological stages, patient survival analysis, similar gene detection, correlation analysis and dimensionality reduction analysis [17]. The association among LDHA mRNA expression level and survival of NSCLC patients was investigated by data mining in the Kaplan–Meier plotter database (http://kmplot.com), an online database integrating gene expression and survival information simultaneously download from Gene Expression Omnibus (GEO) and The Cancer Genome Atlas (TCGA) [18]. The detailed GEO information included are as follows: CaArray, GSE14814, GSE19188, GSE29013, GSE30219, GSE31210, GSE3141, GSE31908, GSE37745, GSE4573, and GSE50081 [19]. Overall survival (OS) and progression-free survival (PFS) were calculated by the Kaplan–Meier method and compared with log-rank test.

### Gene set enrichment analysis (GSEA)

GSEA analyses of lung adenocarcinoma and normal lung tissue samples from the TCGA database were performed to determine the enriched genes. Based on the median expression value, lung adenocarcinoma samples were subdivided into high and low LDHA expression groups, and the functional gene set file “msigdb.v7.1.symbols.gmt” was used to determine the enriched genes. The p value for all the pathways was calculated after 1000 permutations, and pathway enrichment score was obtained in a weighted manner. The pathways and genes with a P-value < 0.05 and false discovery rate (FDR) < 0.25 were considered significantly enriched.

### Cell culture and reagents

All cell lines were purchased from the American Type Culture Collection (ATCC, Manassas, USA), and cultured in DMEM medium (Gibco; Gaithersburg, MD, United States) containing 10% fetal bovine serum, 100 µ/mL penicillin and 100 µg/mL streptomycin at 37 °C under 5% CO_2_. Oxamate sodium (Catalog # O2751) was bought from SigmaAldrich Corp (St. Louis, MO, USA).

### Patient samples and immunohistochemical (IHC) assay

All specimens were collected from NSCLC patients who received radical resection from January 2014 to December 2018 without preoperative treatment in Zhejiang Cancer Hospital. The inclusion criteria included (1) histologically confirmed lung adenocarcinoma (LUAD) or lung squamous carcinoma (LUSC); (2) age was between 20 and 75 years; (3) pathological staging was pT1-T3N1-2M0, according to the AJCC 8th edition; (4) no preoperative treatment was given. Final diagnosis was confirmed by pathological examination. The expression of LDHA in tumor tissues and adjacent normal tissues was determined by IHC analysis. Briefly, paraffin-embedded tissues cut at 4 μm were rehydrated, treated with 3% H_2_O_2_, and blocked with 3% BSA. Subsequently, the tissue sections were incubated with anti-LDHA antibody (1:200, Catalog No. ab101562, Abcam Company, USA) or with control IgG overnight at 4 °C. The immunoreactivity of LDHA was evaluated by the LDHA histo-score, calculated by adding the score for the percentage of positive cells (0–100%) and the staining intensity score (0: no staining, 1: weak, 2: moderate, and 3: strong). Tissues with immunoreactivity scores of 0–4 were considered as low expression, and those with scores ≥ 4 were designated as high expression. All sections were evaluated by two independent pathologists.

### Colony formation assay

Cells treated with 20 mM oxamate or negative control were incubated for 48 h and then were subjected to a range doses of irradiation (0, 0.5, 1, 3, 6, 9 Gy). Next, the cells were incubated at 37 °C in 5% CO_2_. Two weeks later, colonies were washed twice with PBS, fixed with methanol for 30 min, and stained with crystal violet. Colonies with ≥ 50 cells were counted. Plating efficiency (PE) was calculated as colony number/plating cell number) × 100% and the surviving fraction (SF) was equal to colony number/(cells seeded × plating efficiency). The multi-target single-hit model was applied to fit survival curves using the formula: SF = 1 − (1 − e^−D/D0^)^N^, in which D = radiation dose, D_0_ = mean death dose, N = extrapolate number, Dq = quasi-threshold dose. The sensitization enhancing ratio (SER) was calculated as a ratio of D_0_ between treatment and control groups.

### Cell cycle analysis and apoptotic assay

Cells were collected and fixed with 70% pre cooled ethanol overnight, followed by staining with propidium iodide (Catalog No. 537060 10 µg/mL; Sigma-Aldrich) in the dark for 30 min. Flow cytometry was performed on the FACS Calibursystem (Becton Dickinson, CA, USA) and cell cycle distribution was analyzed by means of ModFit LT software (Becton Dickinson, CA, USA). AnnexinV-FITC apoptosis kit (Catalog No. 556547, BD Biosciences, CA, USA) was employed to test apoptosis. Cells were harvested after 24 h irradiation, then stained with AnnexinV/PI for 30 min. The results were analyzed by the FACS Calibur system with ModFit’s LT software.

### Comet assay

Single cell gel electrophoresis (Neutral) was performed with Comet Assay kit (Catalog No. 4250-050-K, Trevigen, Gaithersburg, MD) according to manufacturer’s instructions. Briefly, A549 cells were irradiated at 6 Gy and were collected at 0, 2 and 24 h after irradiation, followed by washing and resuspension in pre-cooling 4 °C PBS. 1–2 × 10^4^ cells were mixed in 1% low fusion point agarose and placed on a slide. The cells were then treated with lysis solution for 30 min, and rinsed in EDTA unwinding solution, and then subjected to alkaline electrophoresis for 30 min (25 V, 300 mA). Then 50 cells per slide were counted and analyzed under fluorescence microscopy (Olympus FV1000). The length of tail, the percentage of DNA in the tail of each comet were measured using CASP software (Version 1.2.3; available at http://casp.sourceforge.net).

### Cellular metabolites detection

A549 and H1975 cells were incubated with oxamate (20 mM) for 24 h followed by 6 Gy irradiation, after 24 h, then cellular metabolite levels were assayed. Lactate concentration in culture medium was tested, while intracellular ATP and pyruvate levels were determined. ATP levels were detected using commercial assay kits (Catalog No. S0026, Beyotime, Haimen, China), according to the manufacturer’s instructions. Results were expressed as a percentage of the control group. Lactate assay kit (Catalog No. A019-1-1) and pyruvate assay kit (Catalog No. A081-1-1) were purchased from Jiancheng Bioengineering Institute (Nanjing, China).

### Statistical analysis

All results were presented as the mean ± SD (standard deviation of the mean) from three independent experiments. Differences between two groups were examined by the Student’s t-test, and ANOVA test was utilized to assess differences between multiple groups. The survival rates were calculated by the Kaplan–Meier method and compared with log-rank test. P-value < 0.05 was considered statistically significant. Statistical analysis was performed using the GraphPad Prism 5.0 and the SPSS 22.0 (SPSS Inc., Chicago, IL, USA) packages.

## Results

### LDHA is up-regulated in NSCLC tissue and associated with high glycolysis level

**Fig. 1:**
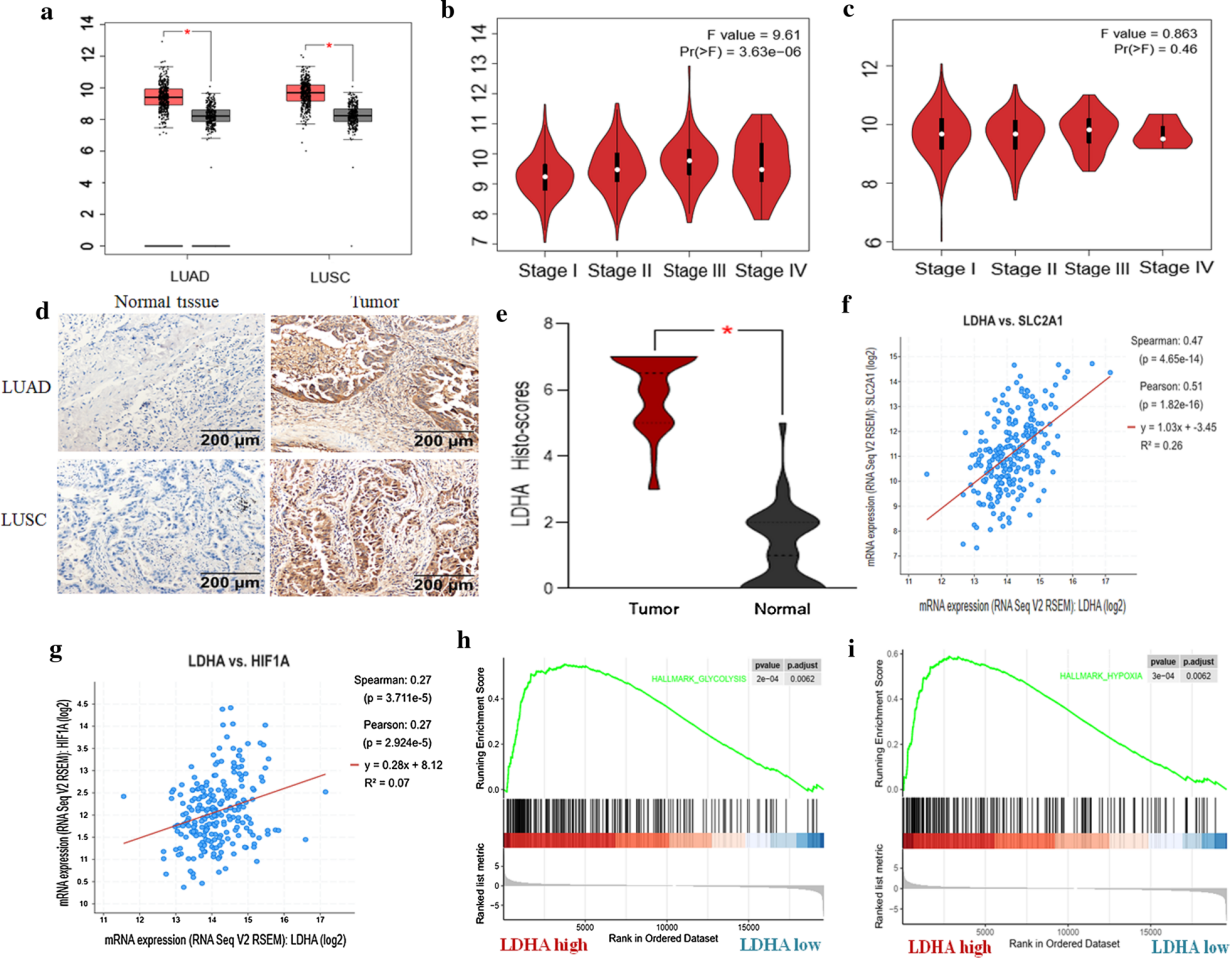
LDHA is up-regulated in NSCLC tissues and correlated with enhanced glycolysis and tumor microenvironment. **a** Using GEPIA browser online, mRNA levels of LDHA are overexpressed in both lung adenocarcinoma (LUAD) and lung squamous carcinoma (LUSC) (p < 0.05). **b** mRNA level of LDHA is positively correlated with tumor stage in patients with LUAD (p < 0.01). **c** LDHA mRNA level is not significantly correlated with tumor stage in patients with LUSC (p = 0.46). **d**, **e** LDHA protein levels are overexpressed in both LUAD and LUSC tissues (35 patients), the histo-scores of LDHA was calculated by adding the score for the percentage of positive cells (0–100%) and the staining intensity scores (p < 0.05). **f**, **g** Using GEPIA browser online, LDHA expression is positively associated with HIF1A and GLUT-1(also named SLC2A1) (p < 0.05). **h**, **i** Using GSEA analysis, high LDHA expression in lung cancer tissues is positively correlated with hallmark gene sets of glycolysis and hypoxia (p < 0.05)

GEPIA browser (http://gepia.cancer-pku.cn/), an online tool for estimating mRNA expression based on The Cancer Genome Atlas (TCGA) and the Genotype-Tissue Expression (GTEx) projects, was employed to compare the mRNA expression level of LDHA in NSCLC tumor and normal tissues. Box and stage plotting analyses were processed on this database. The cutoff p value was defined as 0.01. As showed in Fig. 1a, the mRNA expression levels of LDHA were significantly elevated in both squamous and adenoma of lung cancer. However, the mRNA levels displayed significant correlation with the tumor stage in patients with lung adenocarcinoma (LUAD) instead of lung squamous carcinoma (LUSC) (Fig. 1b, c). To confirm the findings from online database, 35 NSCLC and 20 normal adjacent tissues in our biological sample bank were collected and ICH was performed, the results demonstrated that the protein levels of LDHA were also increased in both LUAD and LUSC tumor tissues, compared to adjacent normal tissues (Fig. 1d, e). Then, we used GEPIA to explore the gene expression correlation between LDHA and glycolysis markers, including GLUT-1 (also named SLC2A1) and HIF1A, which play critical roles in glycolysis and tumor microenvironment. The results showed that LDHA expression was positively associated with both the expressions of HIF1A and SLC2A1 (Fig. 1f, g). In addition, according to GSEA analysis, high expression of LDHA in lung adenocarcinoma tissues was also positively correlated with hallmark gene sets of glycolysis and hypoxia (Fig. 1h, i), indicating the reliability of the results.

### High expression of LDHA is associated with radioresistance and worse survival in NSCLC

**Fig. 2:**
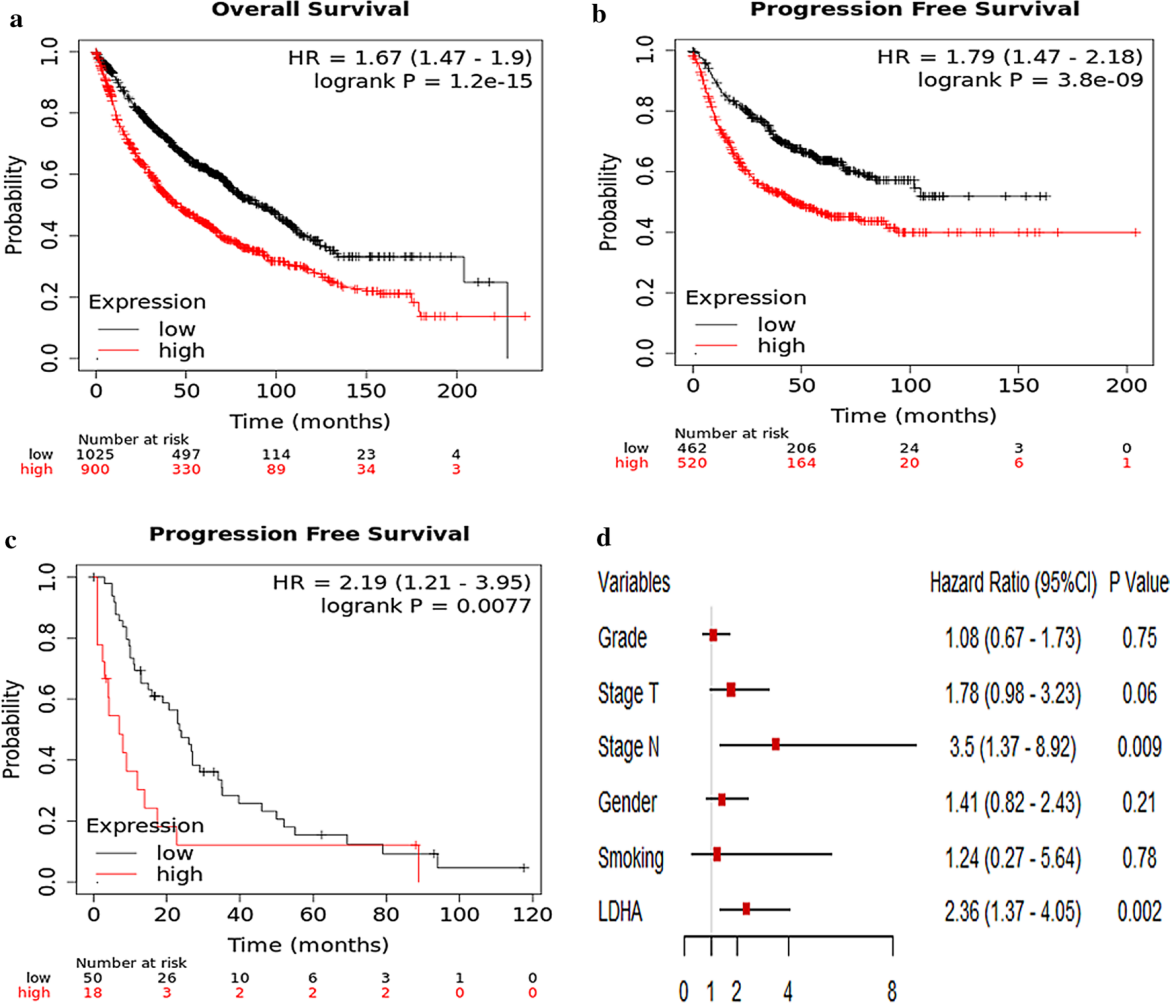
High expression of LDHA is associated with worse survival in NSCLC. Using Kaplan–Meier plotter database online including 1928 NSCLC patients (http://kmplot.com), the association between LDHA expression and lung cancer prognosis is explored, clinical characteristics of the datasets included in the analysis were showed in the Table 1 of the original article [19]. **a** Overall survival (OS); **b** Progression Free survival (PFS); **c** PFS in NSCLC patients received radiotherapy; **d** Forest plots displaying multivariate Cox analysis of OS in NSCLC patients. The control groups were patients with the characteristics of grade 1, stage T1, stage N0, female, no smoking, and low LDHA expression, respectively

To explore the association between the mRNA expression of LDHA and survival in NSCLC patients, Kaplan–Meier plotter database was utilized, which generated gene expression and clinical data from a total of 1928 NSCLC patients, from the following databases: CaArray, GSE14814, GSE19188, GSE29013, GSE30219, GSE31210, GSE3141, GSE31908, GSE37745, GSE4573, and GSE50081. Patient demographic information including age, sex, smoking history, histology, stage, grade, success of surgery, radiotherapy and applied chemotherapy were collected, none of these variables significantly affected data distribution. Clinical characteristics of the datasets included in the analysis was listed in Table 1 in the original article [19]. The cutoff point was automatically selected by the database, to reduce false discovery rate. Overall survival (OS) and progression-free survival (PFS) were calculated using the Kaplan–Meier method and compared with log-rank test. As shown in Fig. 2, the number-at-risk cases, hazard ratios (HRs) with 95% confident intervals (CIs), and P-values were presented, respectively. The results showed that the higher levels of LDHA mRNA expression were associated with worse OS and PFS in the overall population. Multivariate analysis further verified the inverse association between LDHA expression and overall survival of NSCLC, with a HR of 2.36 (95% CI 1.37–4.05, p < 0.05). Especially, in the subgroup analysis, we found that LDHA was also associated with inferior PFS in patients who received radiotherapy, with an HR of 2.19 (1.21–3.95, p < 0.05), even in a relatively small number of samples. Our results preliminarily indicated that high LDHA expression was a poor prognostic factor for NSCLC patients and might be associated with radioresistance.

### LDHA inhibition increased sensitivity of NSCLC cells to irradiation

**Fig. 3:**
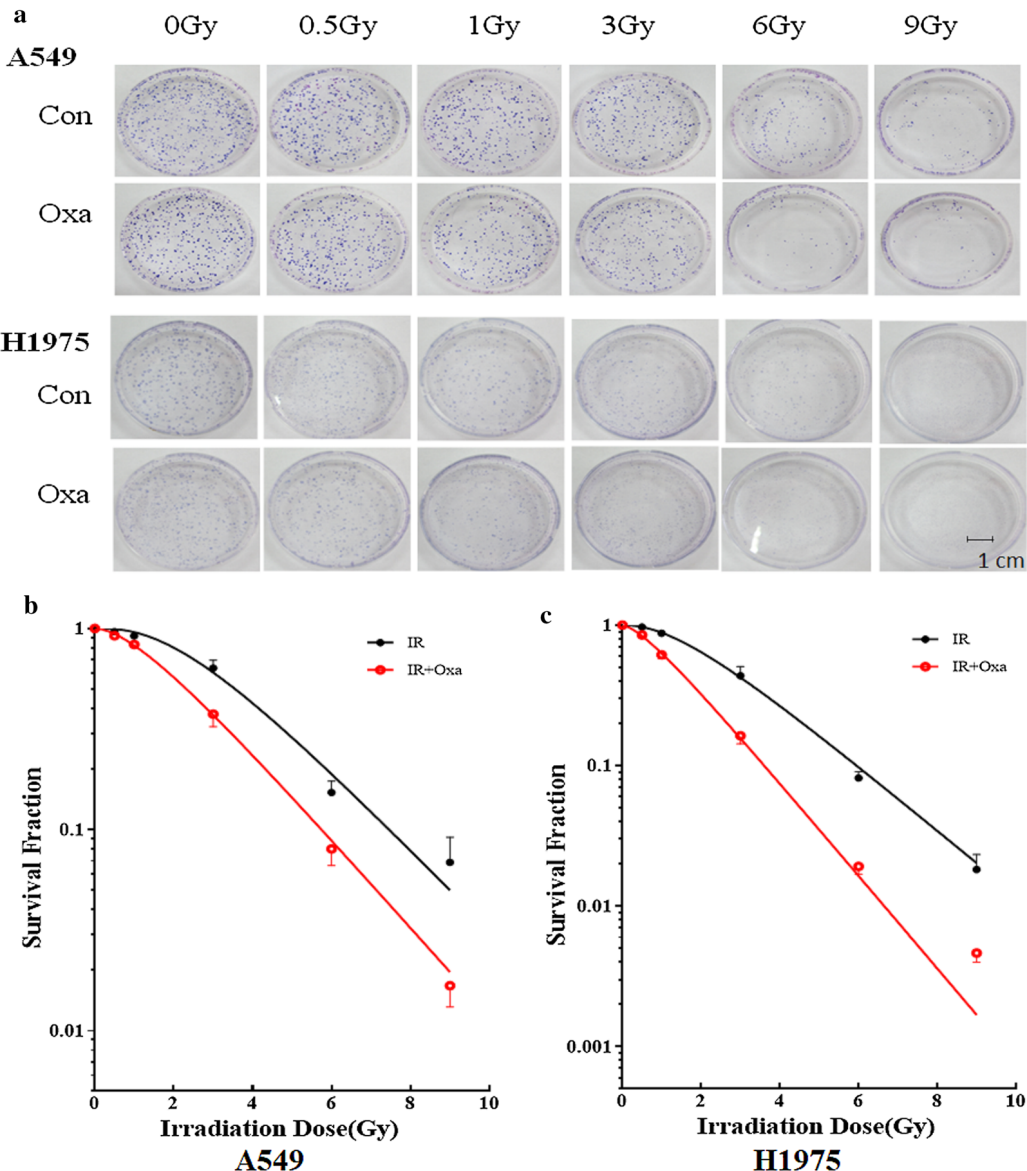
LDHA inhibition by oxamate enhanced the radiosensitivity of A549 and H1975 cells in vitro. **a** Cells pretreated with 20 mM oxamate or negative control were incubated for 48 h and then subjected to a range doses of irradiation (0, 0.5, 1, 3, 6, 9 Gy). Two weeks later, colonies with ≥ 50 cells were counted. **b**, **c** Survival curves were calculated using a multitarget single-hit model. Data represent three independent experiments. *Con* control, *Oxa* oxamate, *IR* irradiation

Colony formation assay was used to examine the impact of LDHA inhibition on the radiosensitivity of NSCLC cell lines in vitro. According to our previous study [16], A549 and H1975 cell lines were pre-treated with or without a low concentration of 20 mM oxamate for 24 h, which exerted negligible influence on cell viability, and then were irradiated with 0, 0.5, 1, 3, 6 and 9 Gy X-ray irradiation, respectively. After incubation for more 2 weeks, the multi-target single-hit model was adopted to fit the survival curves. As shown in Fig. 3, the survival rates were significantly lower in the combined treated groups than cells treated by irradiation alone, and the survival curves decreased in a dose-dependent manner in both A549 and H1975 cells. The sensitivity enhancement ratios (SERs) were 1.10 and 1.43 in A549 and H1975, respectively, indicating that the radiosensitive effect of LDHA inhibition was more effective in H1975 cells.

### LDHA inhibition altered cell cycle distribution after irradiation

**Fig. 4:**
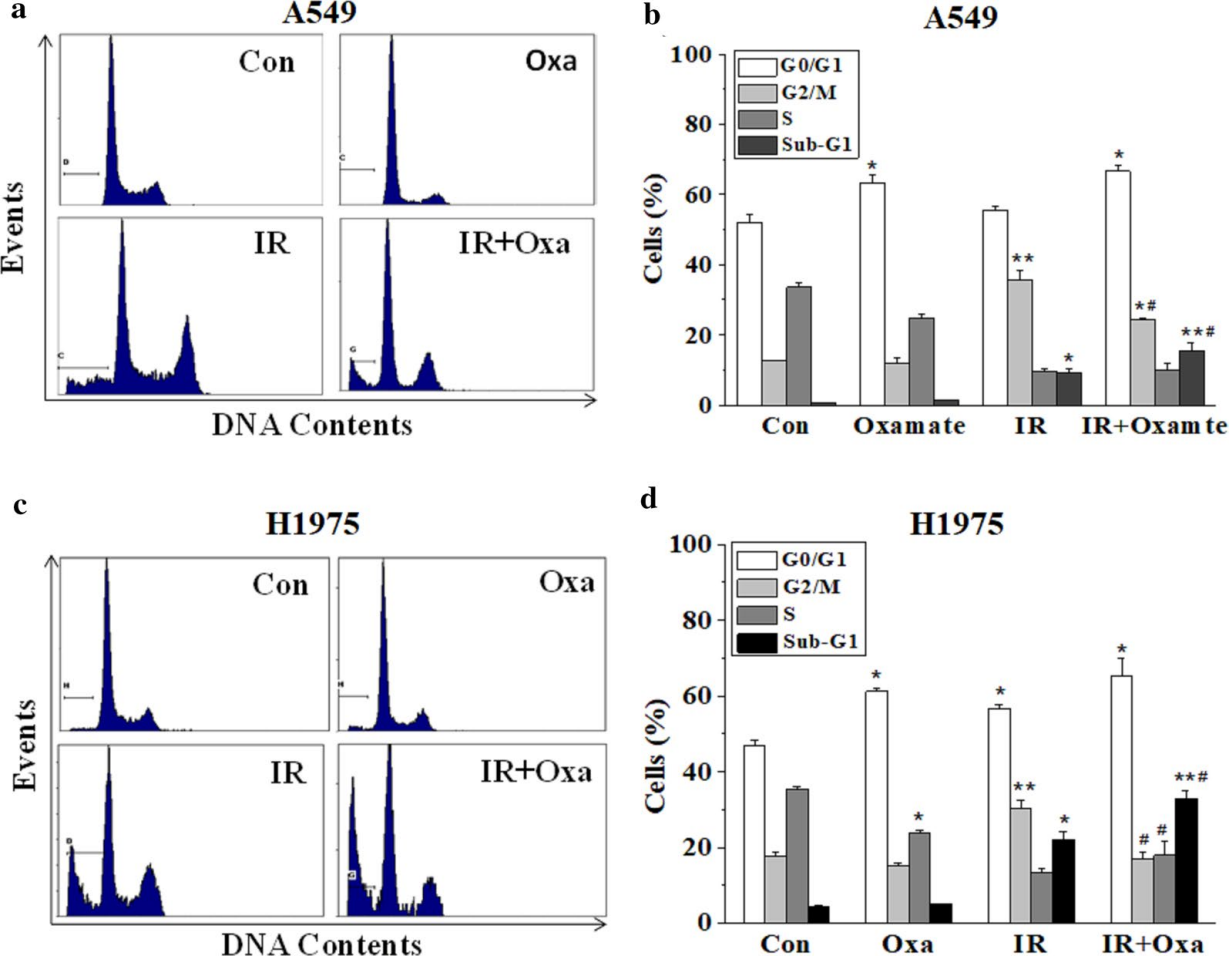
LDHA inhibition alters cell cycle distribution after irradiation. A549 and H1975 cells were pretreated with or without 20 mM oxamate and irradiated with 6 Gy X-ray, after 24 h incubation, flow cytometry was performed to detect alternations in cell cycle distribution. **a**, **b** A549 cells; **c**, **d** H1975 cells. *Con* control, *Oxa* oxamate, *IR* irradiation. Error bars represent ± SD of N = 3 experiments, *p < 0.05, compared to the control group; ^#^p < 0.05, compared to the irradiation group

To further investigate the underlying mechanism of the radiosensitive effect induced by LDHA inhibition, cells were pretreated with or without 20 mM oxamate and irradiated with 6 Gy X-ray, after 24 h incubation, flow cytometry was performed to detect alternations in cell cycle distribution. As shown in Fig. 4, irradiation significantly induced G2/M cell arrest for both NSCLC cell lines while LDHA inhibition led to a substantial increase of cells at G0/G1 phase, accompanied by a reduction of cells at S phase. When combined with irradiation, LDHA inhibition further reduced the ratios of irradiated cells entering G2/M cycle. For instance, the ratios of irradiated cells in G2/M cycle were 43.65 ± 2.80% and 30.25 ± 2.27% in A549 and H1975 respectively, and the ratios dropped to 25.35 ± 0.22% and 16.90 ± 1.66% (p < 0.05) respectively, when combined with 20 mM oxamate pretreatment.

### LDHA inhibition and IR combination induced both apoptosis and autophagy

**Fig. 5:**
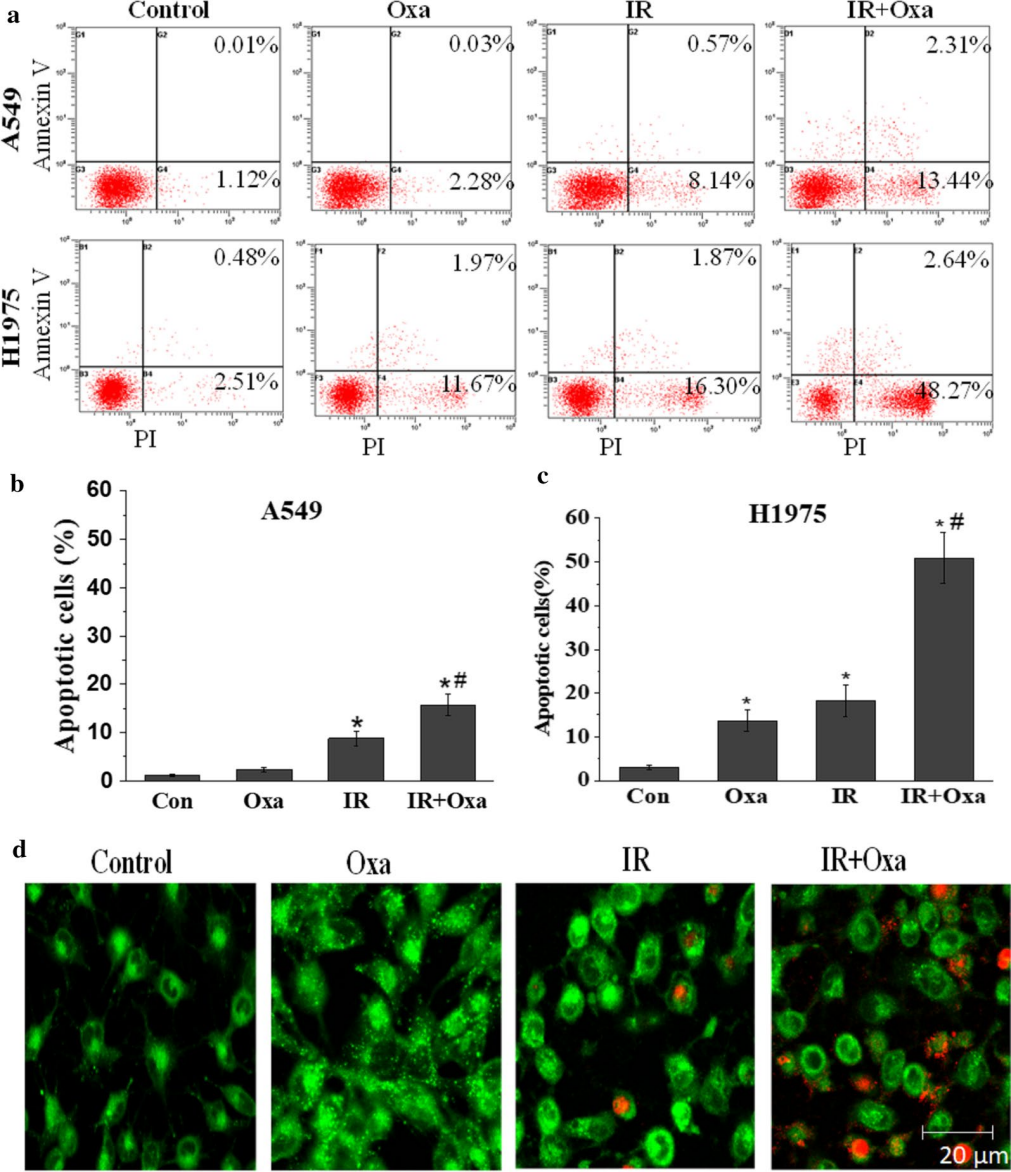
Combination of LDHA inhibition and IR induces both apoptosis and autophagy. **a**–**c** LDHA inhibition significantly promotes cell apoptosis after treatment with 6 Gy X-ray irradiation for 24 h. **d** Using MD/PI double staining, autophagy was detected in A549 cells after oxamate treatment, and interacted with apoptosis induced by irradiation. Autophagosomes appear bright green fluorescence and apoptotic nucleus appear red fluorescence. Error bars represent ± SD of N = 3 experiments, *p < 0.05, compared to the control group; ^#^p < 0.05, compared to the irradiation group

Apoptosis is one of the main biological effects triggered by both LDHA inhibition and irradiation [20, 21]. Therefore, we examined whether LDHA inhibition influenced the IR-induced cell apoptosis. Cells were pretreated with 20 mM oxamate for 24 h and then received 6 Gy irradiation of X-ray. Subsequently, AnnexinV/PI double staining assay was employed to detect cell apoptosis. As shown in Fig. 5a–c, significant apoptosis was observed in H1975 cells after oxamate treatment, while little cells underwent apoptosis in A549 cells, consistent with our previous findings [16]. Importantly, pretreatment with oxamate remarkably enhanced cell apoptosis in both X-ray irradiated A549 and H1975 cells. To explore other potential mechanism in A549 cells, MD/PI double staining was used to detect autophagy in A549 cells after oxamate treatment. We observed that oxamate stimulated autophagic vacuoles and interacted with apoptosis induced by irradiation in A549 cells (Fig. 5d). Interestingly, similar phenomenon was also found for cell treated with combined targeting pyruvate kinase M2 with irradiation in A549 cells [22].

### LDHA inhibition blocked cellular energy metabolism and increased X-ray induced DNA injury through ROS production

**Fig. 6:**
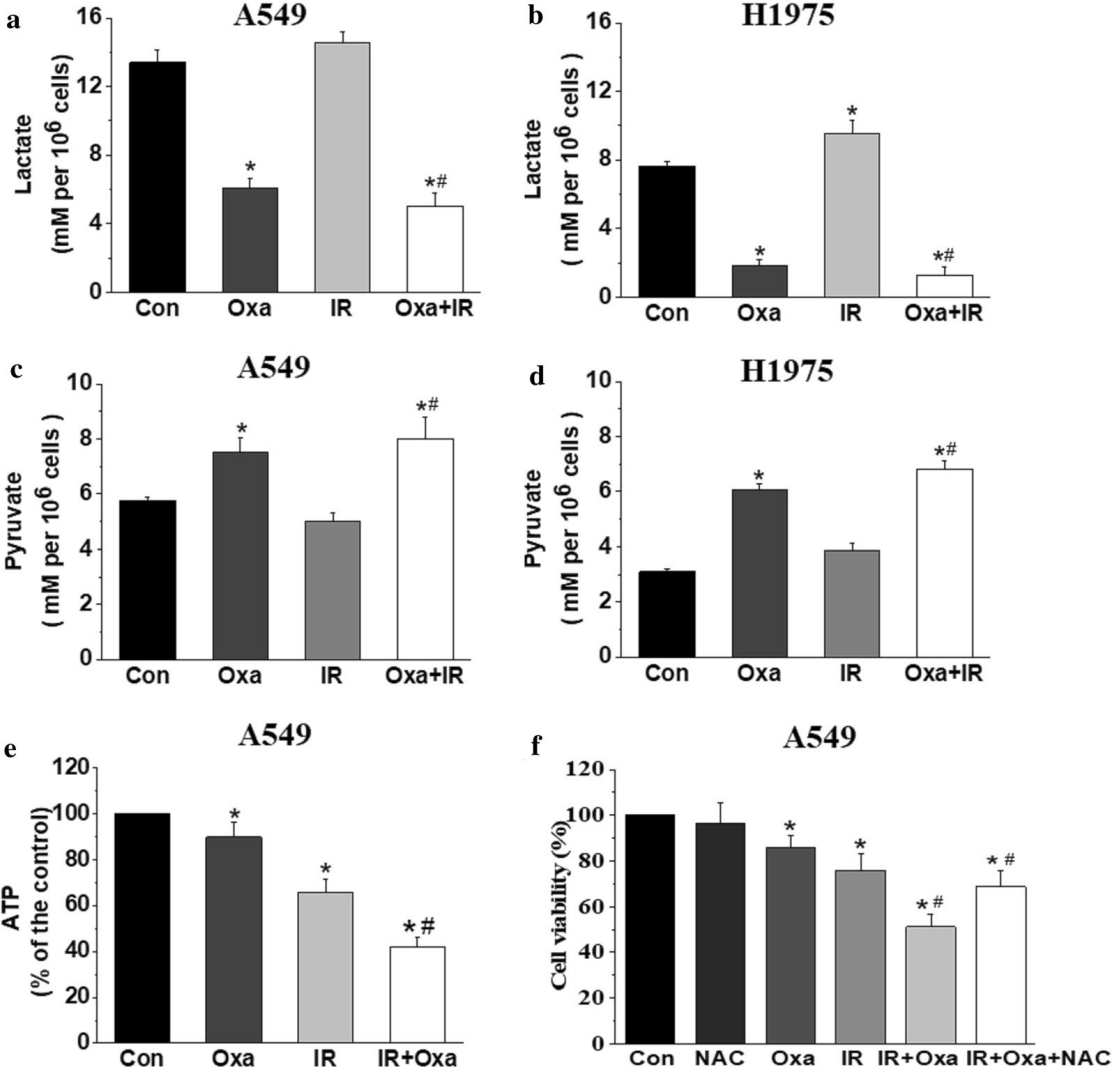
LDHA inhibition blocked cellular energy metabolism in NSCLC. **a**, **b** A549 and H1975 cells were incubated with oxamate (20 mM) for 24 h followed by 6 Gy irradiation, after 24 h, the lactic acid levels in culture medium was tested in each group. **c**, **d** Under the above experiment conditions, intracellular pyruvate levels were tested in A549 and H1975 cells. **e** Intracellular ATP contents were detected in 549 cells. Cells were incubated with oxamate (20 mM) for 24 h followed by 6 Gy irradiation, after 24 h, ATP levels were assayed, and results were expressed as a percentage of the control group. **f** *N*-acetylcysteine (NAC) partly reversed the inhibitive effect of oxamate combined with irradiation. *p < 0.05, compared to the control group; ^#^p < 0.05, compared to the irradiation group

**Fig. 7:**
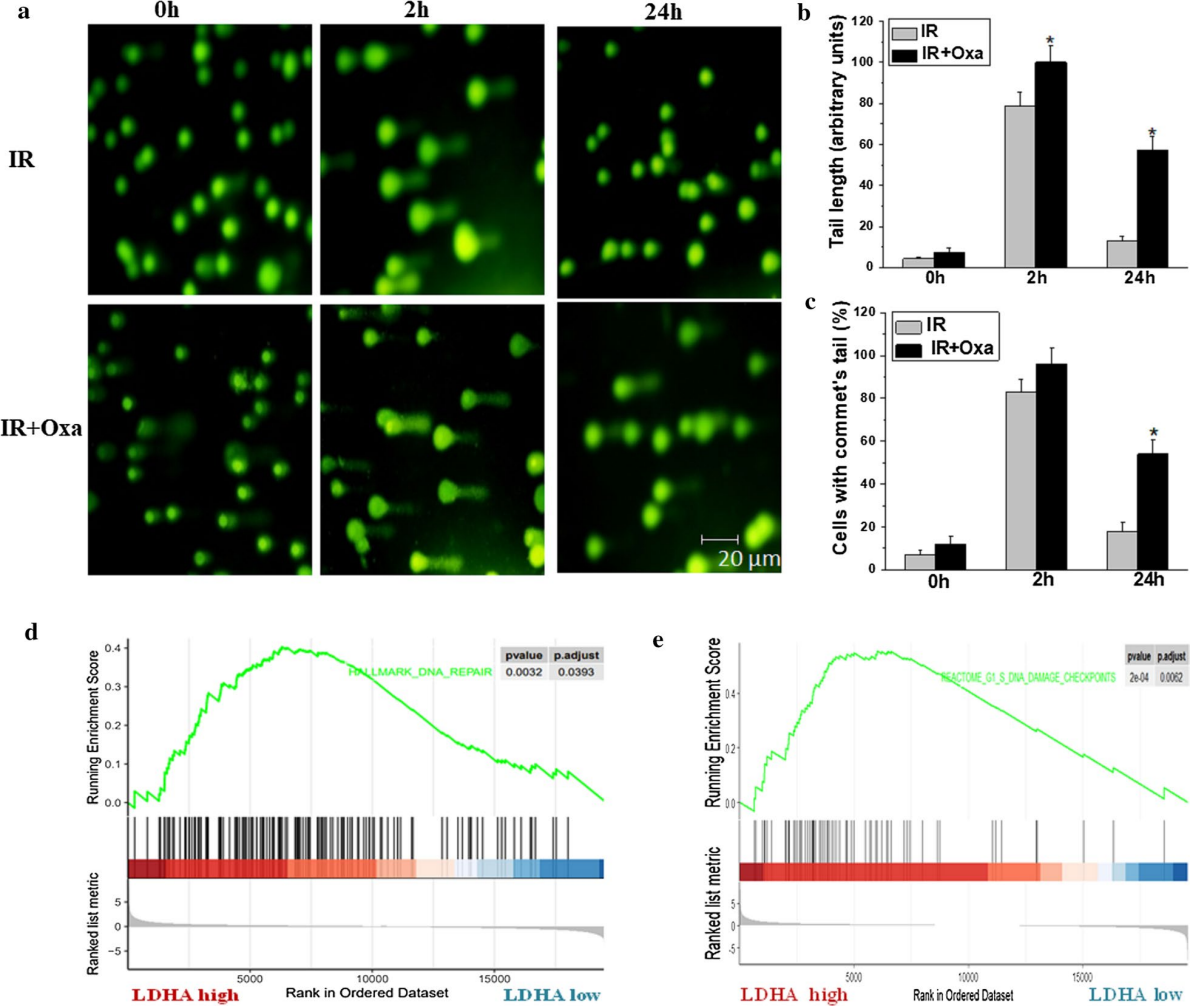
LDHA inhibition increases DNA injury through ROS production. **a** A549 cells pretreated with 20 mM oxmate were irradiated at 6 Gy and collected at 0, 2 and 24 h after irradiation, DNA damage detected by the comet assay. **b**, **c** Oxamate increased both comet tail length and percentage of DNA in the tail significantly after irradiation, 50 cells in each group were counted and analyzed. **d**, **e** LDHA expression was associated with DNA repair capability, as well as G1/S DNA Damage Checkpoints signaling pathway. *p < 0.05, compared to the control group; ^#^p < 0.05, compared to the irradiation group

It is well known that ionizing radiation causes nuclear DNA damage, largely mediated by ROS, and relevant DNA repair mechanisms will also be initiated responding to irradiation, all these factors contribute to the modulation of cell radiosensitivity [23]. As shown in Fig. 6, we found that lactate levels were significantly decreased after oxamate treatment, and slightly increased after irradiation. In contrast, intracellular pyruvate levels were accumulated after LDHA inhibition, and irradiation did not influence the pyruvate levels significantly. Similar results of cellular energy metabolism blockade were observed in both A549 and H1975 cells. Furthermore, we found that the combination of oxamate and IR decreased intracellular ATP concentration significantly in A549 cells (Fig. 6e). As reported previously [20], LDHA inhibition induced accumulation of ROS via mitochondrial oxidative phosphorylation pathway, which might increase DNA damage. To prove this hypothesis, *N*-acetylcysteine (NAC), a thiol antioxidant, was used to pretreat A549 cells with oxamate together. As shown in Fig. 6f, NAC partly reversed the cytotoxicity triggered by combination of oxamate with irradiation, suggesting that ROS induced by LDHA inhibition was involved in the synergistic effect with irradiation. To achieve the modulation of cell radiosensitivity, we next explored whether LDHA inhibition influenced DNA injury using a COMET assay (single cell gel electrophoresis), which provided a qualitative method to measure DNA damage by testing tails of fragmented DNA behind cell nuclei [24]. As shown in Fig. 7a–c, oxamate significantly increased both comet tail length and percentage of DNA in the tail initiated by irradiation, indicating that LDHA inhibition slowed the kinetics of X-ray-induced DSB repair. GSEA analysis also supported that LDHA expression was associated with DNA repair capability, as well as G1/S DNA damage checkpoints signaling pathway (Fig. 7d, e).

## Discussion

Quite a number of lung cancer patients undergo tumor recurrence and metastasis after radiotherapy, and almost one half of loco-regional failure occur within the radiation field, indicating that radioresistance widely exists in NSCLC patients [25]. Many factors might be involved in the poor response to radiotherapy, including hypoxia, cancer stem cell phenotype, cell cycle redistribution and activated DNA repair ability [26, 27]. Cancer cells exhibited higher glucose intake and enhanced glycolysis, to acquire energy and nutrients rapidly, known as Warburg effect [28]. LDHA also known as LDH5, is a key enzyme involving in the glycolysis, and play a vital role in tumor initiation, maintenance, progression, and metastasis [13]. LDHA is reported to be up-regulated in many kinds of cancer, and associated with poor prognosis, including breast cancer [29], colorectal cancer [7], liver cancer [30], bladder cancer [8] and prostate cancer [5, 9], etc. Specially, in the era of immune therapy, serum LDH levels seem also to be associated with the efficiency of PD1/PDL1 efficiency [31].

In this study, we found that LDHA is up-regulated in NSCLC cells using online data, which is also verified by IHC in tumor tissues. Then, we further confirmed that LDHA overexpression was associated with worse survival in NSCLC patients and those received radiotherapy. Using GESA analysis, LDHA expression is positively related to enhanced glucose intake and glycolysis, which promote cancer cell growth and progression. In fact, cancer cell mitochondria and metabolism, also take part in mediating the response to irradiation in tumor, as they regulate multiple processes involved in DNA damage and repair [32]. LDHA overexpression also remodels tumor microenvironment with activated HIF-1 signaling pathways, which is associated with resistance to radiotherapy and results in poorer clinical outcomes [27, 33]. Therefore, LDHA up-regulation in NSCLC leads to increased levels of glycolysis and hypoxic microenvironment, which further contribute to development of resistance to radiotherapy.

The mechanism underlying the radiosensitive effect of LDHA inhibition might involve multiple biological processes, such as ROS accumulation, cell cycle redistribution, increased DNA damage and DNA repair repression. As we know, LDHA inhibition drives cancer cells metabolism from glycolysis to mitochondrial respiration, which results in enhanced oxygen consumption and increased mitochondrial ROS production [20]. High levels of ROS not only induce cell apoptosis or autophagy, but also significantly lead to DNA injury and influence DNA damage response [23]. In our study, the synergistic effect of LDHA inhibition and irradiation are compensated to some extent after NAC treatment, indicating that ROS played a pivotal role in the effect.

Generally, DNA damage repair efficiency is considered as a major determinant of cell radiosensitivity. Specially, double-strand breaks (DSBs) tend to trigger genomic instability and always induce cell-killing effects [34]. In our study, reduced capacity of DNA repair was observed by COMET assay when LDHA expression was inhibited, and further theoretically verified using GSEA analysis. According to previous studies [35, 36], the process might be mediated by PI3K-Akt signaling pathway, which both involves in metabolic reprogramming and tumor cell responsiveness to radiation. Consequently, up-regulation of PI3K-Akt signaling pathway results in acquired radioresistance by enhancing aerobic glycolysis [37]. In fact, PI3K-Akt signaling pathway was indeed down-regulated in A549 cells treated with oxamate in our earlier study [38]. In addition, LDHA controls a most potent pathway of rapid ATP production in cancer cells and its blockage deprives cancer cells from a major energy pathway, which may shift cell metabolism from glycolysis to mitochondrial oxidative phosphorylation (OXPHOS) and generate more ROS [39]. Combination of oxamate with radiation becomes more efficient in suppressing ATP formation and reducing DNA repair ability [40, 41]. Of particular interest, one recent study revealed that tumor metabolites, including 2-hydroxyglutarate, fumarate hydratase, and α-ketoglutarate, hindered DNA repair by disrupting local chromatin signaling [42], demonstrating the importance of metabolism in DNA repair activity. Yet, the molecular crosstalk between glycolysis and DNA repair still needs to be further explored.

As for cell cycle distribution, we found that in both A549 and H1975 cells, after oxamate pretreatment, more cells entered G0/G1 cell cycle and fewer cells were found at S phase, while the ratios of cells at G2/M phase were not significantly influenced. As reported by our previous study, G0/G1 arrest was dependent on the activation of GSK-3β, accompanied by the inhibition of PI3K-Akt pathway [16]. Generally speaking, cells at S phase are the most resistant to radiation, while cells at G2/M phase are the most radiosenstive, and G0/G1 redistribution usually favors radioresistance [43]. Thus, the reduction of cells at radiation-resistant S phase might contribute to increased radiosensitivity in our study. Moreover, using GSEA analysis, we found that LDHA inhibition was associated with inactivation of G1/S DNA Damage Checkpoints signaling pathway [44], which further validated our hypothesis. Similarly, glycolysis inhibitor dichloroacetate was also reported to induce G0/G1 arrest and increase cell sensitivity to the X-ray irradiation in A549 cells [45, 46]. Of note, G2/M arrest was also observed in other cancer cell lines exposed to glycolysis inhibitors and reported to be associated with increased cell radiosensitivity [16, 47]. Therefore, the role of cell cycle re-distribution induced by glycolysis inhibition in modulation of radiosensitivity is still unclear, it’s might be an accompanying consequence, instead of upstream signaling initiation, and less critical than the sensitizing effects of energy deprivation [48].

**Fig. 8:**
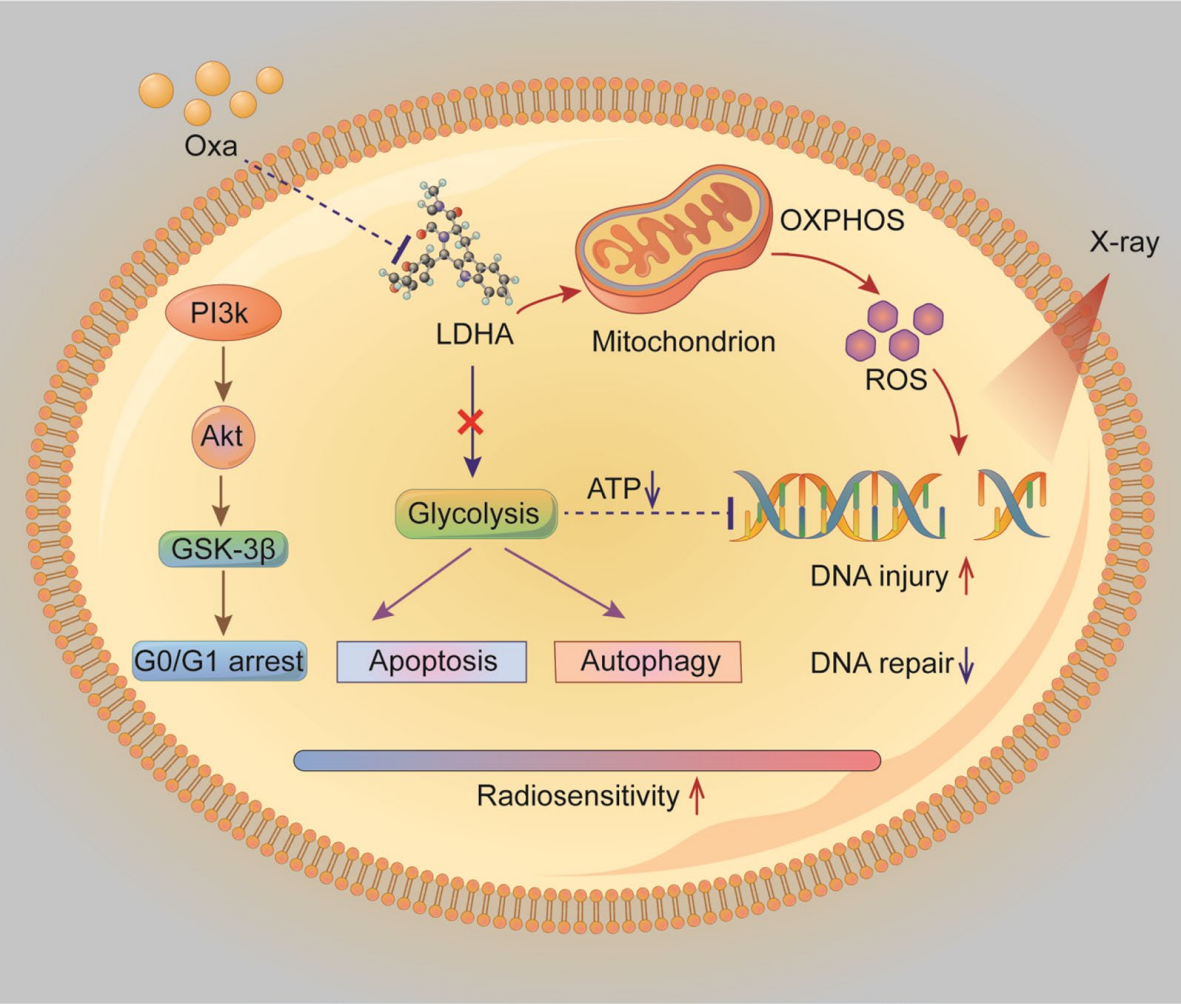
Schematic illustration of the effects observed in this study. LDHA inhibition by oxmate suppressed glycolysis and reduced cellular ATP levels, shifted cell metabolism from glycolysis to mitochondrial oxidative phosphorylation (OXPHOS) and generated more ROS, which might increase DNA injury and hinder DNA repair activity. LDHA inhibition also induced cell apoptosis and autophagy accompanied by cell cycle alternations. All these factors contributed to enhanced radiosensitivity in NSCLC cells

With the deepening of the exploration and development in the field of cancer metabolism, several papers have focused on the effects of altering energy metabolism, such as targeting LDHA or other enzymes involved in glycolysis, on the radiosensitivity of different cancer cells, including cervical cancer [12, 47], glioblastoma [49], head and neck cancer [11, 50]. For instance, dichloroacetate is reported to increase the X-ray sensitivity of human A549 and H1299 NSCLC cells via attenuating aerobic glycolysis and inhibiting DNA double-strand break repair [45]. Tang et al. have recently systematically reviewed the role of metabolism in cancer cell radioresistance and provided a theoretical basis for the improvement of radiotherapy efficiency [51]. Compared to previous studies, this manuscript provides clinical evidence that LDHA overexpression is highly associated with radioresistance and worse survival in NSCLC, and LDHA inhibition by oxamate suppressed glycolysis and reduced cellular ATP levels, shifted cell metabolism from glycolysis to mitochondrial oxidative phosphorylation (OXPHOS) and generated more ROS, which might increase DNA injury and hinder DNA repair activity. LDHA inhibition also induced cell apoptosis and autophagy accompanied by cell cycle alternations. All these factors contributed to enhanced radiosensitivity in NSCLC cells (Fig. 8). This work emphasized the importance of identification of predictive biomarkers and translational significance from bedside to bench.

Of note, although inhibition of LDHA mainly induces cell apoptosis by generating ROS, different responses to glycolysis inhibition were observed in different cancer cells [16, 52, 53]. Many efforts are being made to identify biomarkers to predict the sensitivity of glycolysis inhibitors (including LDHA inhibitors), several genetic mutations have been reported to be associated with the efficiency of glycolysis inhibitors, including Kras [54], PI3K [55], AMPK-mTOR [56],and mitochondrial DNA (mtDNA) mutations [57]. Our results showed the A549 cells with Kras mutation was less sensitive to the combination of LDHA inhibition and radiotherapy than H1975, reflecting the heterogeneity of lung cancer. In addition, it’s recently reported that only a part of NSCLC cell lines may benefit from the combination of radiotherapy and metabolic inhibition [58]. Thus, the radiosensitive effects of LDHA inhibition seems to be different in different cancer cells, efforts are still needed to identify predictive biomarkers and tested by clinical trials.

## Conclusions

In this translational study, we found that the expression of LDHA, no matter mRNA or protein levels, was up-regulated in both lung adenoma and squamous cells. Moreover, higher LDHA expression is associated with enhanced glycolysis, hypoxia microenvironment, radioresistance and worse survival in NSCLC. Targeting LDHA might increase radiosensitivity by enhancing DNA damage through generation of ROS and inhibition of DNA repair by energy deprivation, which give implications for further clinical study and novel drug development for NSCLC.

## Data Availability

The datasets supporting the conclusions of this article are included within the article.

## Abbreviations

NSCLC: Non-small cell lung cancer
LDHA: Lactate dehydrogenase A
RT: Radiotherapy
ROS: Reactive oxygen species
GSEA: Gene set enrichment analysis
LUAD: Lung adenocarcinoma
LUSC: Lung squamous carcinoma
HR: Hazard ratios
OS: Overall survival
PFS: Progression-free survival
OXPHOS: Oxidative phosphorylation
